# Do we have (in)compatibilist phenomenology of deliberation?: a survey

**DOI:** 10.3389/fpsyg.2025.1605079

**Published:** 2026-02-16

**Authors:** Souichiro Honma, Kiichi Inarimori, Kengo Miyazono

**Affiliations:** 1 Laboratory of Philosophy and Ethics, Department of Philosophy and Religious Studies, Graduate School of Humanities and Human Sciences, Hokkaido University, Sapporo, Japan; 2 Uehiro Division for Applied Ethics, Graduate School of Humanities and Social Sciences, Hiroshima University, Hiroshima, Japan; 3 Center for Human Nature, Artificial Intelligence, and Neuroscience, Hokkaido University, Sapporo, Japan

**Keywords:** compatibilism, deliberation, experimental philosophy, free will, incompatibilism, leeway freedom, phenomenology, source freedom

## Abstract

In this article, we survey contemporary works on the phenomenology of deliberation and discuss its relevance to the philosophical problems of free will. We first articulate the debate between compatibilist and incompatibilist interpretations of (leeway and source) freedom in the phenomenology of deliberation (Section 2). Next, we examine this issue in light of relevant psychological studies of the folk experiences of deliberation (Section 3), followed by a discussion of four general methodological problems in the investigation of the phenomenology of deliberation (Section 4): (i) the problem of heterogeneity, (ii) the problem of cognitive penetration, (iii) the problem of reliability, and (iv) the problem of relevance. We conclude that these problems suggest the need for the reconsideration of experimental methods and the role of the phenomenology of deliberation in the free will debate.

## 1 Introduction

Imagine a situation in which you deliberate whether to buy a new book on sale. First, you focus on the book and set aside irrelevant matters, such as how old you are, what time it is, what you had for lunch, and so on. Second, you consider the relevant factors in buying the book, such as the price, the budget, the currency (in the case of buying a foreign book), the extent of interest, and so on. And lastly, you decide whether or not to buy the book. These are the typical events that occur in the process of deliberation. More generally, we take deliberation^1^ to be the process of (i) excluding minor alternatives in order to reduce unnecessary mental activity, (ii) trying to figure out the reasons for favoring or disfavoring options, and (iii) trying to decide the option to be chosen.^2^

The process of deliberation seems to have a specific type of phenomenology.^3^ It seems to make sense to talk about the phenomenology of deliberation, the experience of deliberation, or what it is like to deliberate.^4^ Then, we have some questions called “analytical questions” by (Bayne 2017, p. 633). What is the phenomenology of deliberation, exactly? What are the contents of the experience of deliberation, if it has?

Here is a somewhat lengthy description of the experience of deliberation by O'Connor.

A natural way of characterizing our typical experience of making decisions and acting upon them—one that would, I think, gain widespread assent—goes something like this: When I decide, say, to go for a walk on a cool autumn evening, I am conscious of various factors at work (some consciously articulated, some not) motivating me either to do so or to do something else instead. And there are some courses of action which, while it is *conceivable* that I might choose to follow them, are such that they do not represent “genuine” possibilities for me at that time, given my current mood, particular desires and beliefs, and, in some cases, long-standing intentions of a general sort. But within the framework of possibilities (and perhaps even relative likelihoods) that these present conative cognitive factors set, it seems for all the world to be *up to me* to decide which particular action I will undertake. The decision I make is no mere vector sum of internal and external forces acting upon me during the process of deliberation (if, indeed, I deliberate at all). Rather, *I* bring it about—directly you might say—in response to the various considerations: I am the source of my own activity, not merely in a relative sense as the most proximate and salient locus of an unbroken chain of causal transactions leading up to this event, but fundamentally, in a way not prefigured by what has gone before. Or, again, so it seems. (O'Connor, 1995[2003], p. 257: Emphasis in the original)

O'Connor's description suggests that the phenomenology of deliberation involves at least two things: the sense that we can do otherwise (i.e., we have leeway freedom) and the sense that we ourselves bring about or produce the action (i.e., we have source freedom).

It is expected that an examination of the phenomenology of deliberation can be informative and useful for philosophical debates on free will in general, including the debate about the nature of free will and whether free will is compatible with determinism or not. In other words, it can contribute to addressing “conceptual questions” raised by (Deery and Nahmias 2023), p. 417): how is free will supposed to be defined or analyzed? For a philosophical explication of free will needs to respect the commonsensical understanding of free will that is shared not only by philosophers but also by non-philosophers. Like causation, color, or justice, free will—or practices called the exercises of free will—is a familiar notion in ordinary life. Thus, any attempt to explicate free will must take into account our everyday understanding of it. It is true that our commonsensical understanding is not always a reliable guide to what actually exists, but it has authority in determining which actual processes deserve to be called “free will” (cf. Nolan, 2009, pp. 267–269, p.287). Moreover, our ordinary understanding of free will is arguably shaped by our experience of deliberation (cf. Bayne, 2017, pp. 635–636; Deery and Nahmias, 2023, p. 418).^5^ This is because the deliberative process of deciding which action to perform among multiple options can be regarded as a paradigmatic instance of the exercise of free will.

For example, if it turns out that leeway and source freedom in the experience of deliberation are incompatible with determinism, then this gives a prima facie reason to favor the incompatibilist understanding of free will. Again, if leeway and source freedom are necessary components of the phenomenology of deliberation, then it is expected that the idea of leeway and source freedoms would be deeply entrenched in our commonsensical understanding of free will such that abandoning the idea (and accepting skepticism, eliminativism, or revisionism about free will) might be practically impossible even if we have excellent reasons to do so. Since the ordinary understanding of free will influenced by the phenomenology of deliberation constrains the theoretical range of what free will is, investigating the phenomenology of deliberation impacts our theories of free will. Admittedly, folk phenomenology of deliberation may not play a substantial role due to the practical difficulty of identifying it or the complex nature of the experience itself. However, this does not diminish its theoretical importance. We will return to this point in Section 4.

Our central aim of this review article is to examine contemporary works on the phenomenology of deliberation and evaluate its relevance to the philosophical problems of free will. In Section 2, in order to provide the conceptual background used in psychological studies, we look at the debate between compatibilist/incompatibilist interpretations of leeway and source freedom in the phenomenology of deliberation and explain the major interpretations of these freedoms. In Section 3, we examine this issue, taking into account relevant experimental studies on the folk experience of deliberation. Also, we consider the limits of these researches. In Section 4, we discuss four general methodological problems in the current study of the phenomenology of deliberation: (i) the problem of heterogeneity, (ii) the problem of cognitive penetration, (iii) the problem of reliability, and (iv) the problem of relevance. In conclusion, we suggest that the nature of the phenomenology of deliberation is not easily tractable and theorizable, and thus it is vital to address how we investigate the phenomenology of deliberation prior to appealing to it as a solution to the philosophical problem of free will.

## 2 Leeway and source views

In this section, we review the philosophical debates concerning the phenomenology of deliberation and introduce two major views about the phenomenology of deliberation: the leeway view and the source view. The purpose of this review is to highlight how these debates provide the background concepts or ideas used in the psychological studies to be discussed later in Section 3. Also, examining the philosophical literature is valuable for clarifying intricate distinctions and points at issue. Thus, this review is not intended as a definitive verdict but rather as an illustrative examination of possible theoretical options. According to the leeway view, the phenomenology of deliberation involves the phenomenology of leeway freedom, i.e., the experience of “I can do otherwise,” while according to the source view, the phenomenology of deliberation involves the phenomenology of source freedom, i.e., the experience of “I myself bring about action.” For each view, there is a further question as to whether the content of the phenomenology of deliberation, understood either in the leeway sense or the source sense, is veridical under determinism.^6^

### 2.1 The leeway view

When you deliberate whether you will buy a new book, you take it for granted that you can choose both options, i.e., the option of buying the book and the option of not buying it. If not, you do not consider these options seriously. It is widely accepted that we believe in leeway freedom when we deliberate multiple options (cf. Campbell, 1951, p. 463; Lehrer, 1960, p. 145; Taylor, 1964, p. 74; Castañeda, 1975, p. 134; Van Inwagen, 1983, p. 155; Kapitan, 1986, p. 230; Strawson, 1986[2010], p. 116; Clarke, 1992, p. 101; Searle, 2001, p. 15; Coffman and Warfield, 2005, p. 26; Nelkin, 2011, p. 146; Pereboom, 2014, p. 113). The leeway view concerning the phenomenology of deliberation might be regarded as a variant of this belief.

The leeway view states that we experience leeway freedom when we deliberate.^7^ There are compatibilist and incompatibilist interpretations of the experienced leeway freedom. According to the incompatibilist interpretation, leeway freedom in the experience of deliberation is incompatible with determinism in the sense that, if determinism is true, the content of the experience of deliberation is not veridical; i.e., its content does not represent reality as it is. Here is a typical incompatibilist description of the phenomenology of deliberation:

Reflect very carefully on the character of the experience you have as you engage in normal, everyday ordinary human actions. You will sense the possibility of alternative courses of action built into these experiences. [...] In normal behavior, each thing we do carries the conviction, valid or invalid, that we could be doing something else right here and now, that is, all other conditions remaining the same. (Searle, 1984, p. 95)

The incompatibilist interpretation of experienced leeway freedom is coherent with the standard philosophical understanding of leeway freedom, understood as the ability to do otherwise under exactly the same conditions, which is incompatible with determinism. Determinism is the thesis that necessarily, if the entire state of the world at some past time and the laws of nature are given, then subsequent states of the world are fixed.^8^ Suppose that every condition, including the entire past state of the world and the laws of nature, is specified. Also, suppose that we cannot do anything to change the past state, the laws of nature, and their consequences. It follows from these assumptions and determinism that the action we perform, which is considered as the consequence of the past state and the laws of nature, is fixed, and thus there is no alternative action we can perform. Therefore, the ability to do otherwise in the case where the background conditions are exactly the same is incompatible with determinism.^9^

According to the compatibilist interpretation, in contrast, leeway freedom in the phenomenology of deliberation is compatible with determinism in the sense that, even if determinism is true, the content of the experience of deliberation can be veridical. There are two versions of leeway compatibilism in the phenomenology of deliberation. The first version is the classical compatibilist interpretation, which is described in the following quote:

Let us carefully examine the content of the feeling that on a certain occasion we could have acted on other than the way we did, in fact, act. What we did find? Does the feeling we have inform us that we could have acted otherwise *under exactly the same external and internal motivational conditions?* No, says determinist, this feeling simply discloses that we were able to act in accord with our strongest desire at that time, and that we could indeed have acted otherwise if a different motive had prevailed at the time. (Grünbaum, 1952, p. 672: Emphasis in the original)

According to the classical compatibilist interpretation, leeway freedom is succeeding in doing otherwise *if we were to will or try to do otherwise* (cf. Moore, 1912[2005]; Ayer, 1954; Nowell-Smith, 1954). Determinism is perfectly compatible with the leeway freedom in this conditional sense (although it is incompatible with the antecedent of the conditional, assuming the original states of the world and the laws of nature are fixed).

The second version of leeway compatibilism is the epistemic or doxastic interpretation (Deery, 2021; cf. Kapitan, 1986; Nelkin, 2011; Pereboom, 2014). According to this version, the leeway freedom in the experience of deliberation involves the idea that options are epistemically or doxastically open in the sense that these options have not been excluded by our knowledge or belief. For example, when you deliberate whether you buy a book or not, the experience of deliberation involves the sense that both options, i.e., the option of buying the book and the option of not buying it, have not been excluded by your knowledge or belief. Since these epistemic or doxastic possibilities are compatible with determinism (e.g., both options can still be epistemically or doxastically open even if one of the options has been ruled out by determinism as a matter of fact), this interpretation of leeway freedom is a compatibilist one.

These three views—incompatibilist, classical compatibilist, epistemological or doxastic compatibilist views—are the main interpretations of leeway freedom in the phenomenology of deliberation.

### 2.2 The source view

The source view is the thesis that the experience of deliberation involves source freedom. Source freedom in the experience of deliberation, just like leeway freedom, is open to multiple interpretations, including compatibilist ones and incompatibilist ones. Horgan argues that free-agency phenomenology seems to include both leeway and source freedom and characterizes source freedom in that phenomenology as follows^10^:

You experience your arm, hand, and fingers as being moved by you yourself – rather than experiencing their motion either as fortuitously moving just as you want them to move, or passively experiencing them as being caused by your own states. You experience the bodily motion as generated by yourself. (Horgan, 2011, p. 79: Emphasis in the original; cf. Horgan, 2007a,b, 2015)

According to Horgan, the experience of deliberation seems to involve the sense that an action is caused by ourselves, which are distinct from our own mental states. Thus, the source freedom in the above passage is not about event or state causation (where agents cause their actions by their own mental events or states that are causally efficacious), but rather about agent causation (where agents, who are causally efficacious by themselves, cause their actions).^11^

There is a debate as to whether, assuming that the source freedom in the experience of deliberation involves the sense of agent causation, the agent causation in the experienced source freedom is compatibilist (i.e., the content of the experienced source freedom in deliberation can be veridical in a deterministic universe) or incompatibilist (i.e., the content of the experienced source freedom in deliberation cannot be veridical in a deterministic universe). The incompatibilist agent causation interpretation is defended by O'Connor. Recall O'Connor's (1995[2003], p. 257) statement we referred to in the introduction that we are the source of our own activity, not just as the significant locus of the causal chain but also fundamentally not prefigured by what has happened in the past. The source freedom defended by O'Connor is considered as incompatibilist agent causation since it is not predetermined by prior events and we ourselves play a central part in causal chains.

In contrast, Pereboom defends a compatibilist agent causation interpretation of source freedom in the experience of deliberation. According to him, even an agent who does not have the ability to do otherwise can still experience that the formation of her intention is causally determined by her perceiving the environmental factors and her own character (Pereboom, 2015, p. 288). Since she experiences that her action is partially determined by her own character, it is she who causes the formation of the intention. Thus, the experienced source freedom can be considered as a kind of determined agent causation.

There is also a view according to which the source freedom in the experience of deliberation involves event causation rather than agent causation. For example, Bayne suggests that we can experience ourselves as the source of actions without experiencing ourselves the agent cause. This view is compatible with what (Bayne 2017, p. 638) calls “place-holder conceptions of the phenomenology of the self,” according to which the self in the phenomenology functions only as the “location” of mental events which cause our actions when we deliberate. For example, when you deliberate whether you buy a book on sale, the experience of deliberation involves the sense that your mental states, including beliefs and desires, will be causally responsible for your decision and action. Since event causation theory does not require agent causation, this theory is more deflationary in the sense that it avoids introducing redundant entities.

In sum, both the discussion of leeway freedom and that of source freedom in the experience of deliberation are diverse and complex.^12^ Philosophers disagree on several points: whether the leeway view or the source view is correct; whether leeway freedom is compatible with determinism (incompatibilism versus classical or epistemic/doxastic compatibilism); whether source freedom is compatible with determinism; and whether source freedom should be explicated in terms of agent causation or event causation.

## 3 Folk phenomenology

So far, we have discussed philosophers' accounts of leeway and source freedom in the experience of deliberation, which are typically justified by means of introspection, intuitive judgment, or thought experiments about their own experience of deliberation. But, we are not only interested in philosophers' experience of deliberation, but rather in the experience of deliberation in general, including the experience of non-philosophers. What do non-philosophers say about their experience of deliberation? Which philosophical account of the experience of deliberation is coherent with non-philosophers' experience of deliberation? To investigate these issues, we need empirical studies of folk phenomenology of deliberation. We first review the contents and results of these empirical studies, and then discuss their limitations and future directions.

### 3.1 Experiments

Although numerous psychological studies investigate free will, experiments that probe not only the phenomenology of deliberation but also its relation to free will are relatively rare. For example, the well-known experiment conducted by Libet and colleagues does not deal with the phenomenology of deliberation (Libet et al., 1982, 1983; Libet, 1985; cf. Keller and Heckhausen, 1990; Haggard and Eimer, 1999; Trevana and Miller, 2002). In this experiment, six participants performed a simple motor action—“the quick, abrupt flexion of the fingers and/or the wrist” of a hand (Libet et al., 1983, p. 625)—and reported the “W times,” the time of their first awareness of wanting (or wishing or willing) to act, using a clock that completed one rotation every 2,560 ms.^13^ Across forty trials, EEG recordings revealed that neural activity—dubbed the type II readiness potential (RP)—preceded the awareness of the wanting to act. On average, type II RPs occurred about 350–400 ms before the reported W times and 500–550 ms before the actual motor action.

Are the forms of awareness investigated in these experiments identical to the phenomenology of deliberation? No, for deliberation requires at least the process of trying to consider the reasons for favoring or disfavoring options and trying to decide the option to perform (cf. Roskies, 2011, pp. 18–19; Bayne, 2011, pp. 28–30). Indeed, Libet himself stated that “[t]he ‘act now' process should be distinguished from deliberations and advance making of choices about performing an act” (Libet, 2004, p. 132). Rather, these experiments examine—arguably—the phenomenology of decision or intention.^14^ Moreover, these experiments investigate only the “W times,” and thus do not address the content of experience, i.e., whether it contains leeway or source freedom, or whether it can be veridical under determinism.

A similar point applies to the experiments conducted by (Soon et al. 2008, 2013), which investigate the extent to which neural activity precedes choices between among multiple options. Although these experiments probe the process of trying to decide the option among multiple options (e.g., pressing either a left or right button, or performing either addition or subtraction), they do not examine the process of considering reasons for or against those options. Nor do they address what the content of the phenomenology of deliberation is and how it relates to free will. Hence, Libet-style experiments are not regarded as studies that sufficiently investigate the phenomenology of deliberation.

Libet's study is just one example of experimental investigation into the nature of free will. Setting aside Libet's experiment, there are many empirical studies examining whether people acknowledge the existence of free will or ascribe moral responsibility under conditions involving causal determinism (see Björnsson, 2022; Inarimori et al., 2024b for reviews). Still, the focus of these studies has largely been limited to people's intuitions about whether one can be free or morally responsible under deterministic conditions. For example, canonical studies such as (Nahmias et al. 2005, 2006) and (Nichols and Knobe 2007) investigated people's intuitions about free will and moral responsibility under determinism using hypothetical vignettes describing deterministic universes. However, these studies did not examine people's experiences during deliberation. In other words, most work in experimental philosophy on free will has focused on third-person intuitive attributions of free will and moral responsibility, rather than on people's first-person phenomenological experiences of free will.

Let us now review the experiment that investigates the phenomenology of deliberation and its relation to free will. An early attempt to investigate folk experiences of deliberation was the study by Nahmias and colleagues (Nahmias et al., 2004). In their pilot experiment, participants (96 undergraduates who had never studied philosophy of free will) answered the following question (Nahmias et al., 2004, p. 174):

Imagine you've made a tough decision between two alternatives. You've chosen one of them and you think to yourself, “I could have chosen otherwise” (it may help if you can remember a particular example of such a decision you've recently made).

Which of these statements best describes what you have in mind when you think, “I could have chosen otherwise”?

A. “I could have chosen to do otherwise even if everything at the moment of choice had been exactly the same.”
B. “I could have chosen to do otherwise only if something had been different (for instance, different consideration had come to mind as I deliberate or I had experienced different desires at the same time).”
C. Neither of the above describes what I mean.

This process of tough decision between two alternatives can be regarded as deliberation since it includes the process of focusing exclusively on two options, trying to figure out reasons, and trying to decide which option to perform as one would in an actual tough decision. The results were that about two-thirds of participants chose the compatibilist description (B); about one-third chose the incompatibilist description (A); a few chose (C) (Nahmias et al., 2004, pp. 174–175). From these results, Nahmias et al. suggested that non-philosophers experience compatibilist leeway freedom in deliberation.

In contrast, (Deery et al. 2013, pp. 135–139) conducted experiments that focused on the concurrent experience of the ability to do otherwise and included the comprehension test of determinism. For example, in one experiment (study 2), participants were asked to choose between two options for charities. In condition 1, the deliberation was performed only in their imagination. In condition 2, the deliberation was actually performed; i.e., participants were told that real money would be donated in accordance with their decision.^15^

In these conditions, participants were asked to demonstrate their level of agreement with the statement that their phenomenology of deliberation includes leeway freedom on a seven-point scale (1 means disagree completely; 7 means agree completely). This is an example of the statement they used (Deery et al., 2013, p. 138):

When deciding which option to choose, it feels like I can either choose to donate to the endangered tree *Castanea Dentata* or choose to donate to the Childhood Cancer Foundation.

This process is a kind of deliberation, for it is restricted to two particular options and involves the process of trying to consider the reasons for or against them and trying to determine to which charities to donate. Only participants who demonstrated agreement with the leeway statement with a 5 score or higher proceeded to the section of the comprehension test of determinism.

In the comprehension test of determinism section, participants were presented with a definition of causal completeness,^16^ and subsequently provided with a description where causal completeness holds.^17^ The comprehension of determinism was assessed using a questionnaire consisting of two true-or-false questions about the description.^18^ When a participant gave the wrong answer, she was provided with the correct answer and an explanation of it, followed by a similar question to double-check her understanding. Participants who answered two questions correctly, whether the first or second time, were assumed to have a good understanding of determinism and were moved to the next section, which is about the compatibility question.

In the compatibility question section, participants were asked to recall their agreement on their leeway freedom in the first part of the experiment, and answer questions about the compatibility between the experienced leeway freedom and causal completeness. For example, in conditions 1 and 2, participants were asked to show agreement on a 7-point scale with the following statement (where responses of 5 or higher were taken as incompatibilist answers):

Even though it felt like I could either choose to donate to Castanea Dentata or choose to donate Ulmus Dentata, if causal completeness is true then I couldn't really have chosen differently than I did.

The result of this experiment^19^ was that the means of the answers to the compatibility questions in condition 1 was 5.60, *t*_(34)_ = 6.08, *p* < 0.001; the mean of the answers in condition 2 was 5.78, *t*_(36)_ = 6.85, *p* < 0.001 (Deery et al., 2013, pp. 138–139).^20^ This result suggests that the participant's experience of deliberation includes incompatibilist leeway freedom, not only in imaginary deliberations (condition 1) but also in real deliberations (condition 2).

In order to resolve the question of whether the phenomenology of leeway freedom is either compatibilist or incompatibilist, (Deery et al. 2013, pp. 140–143) conducted another experiment (study 3), which examined whether ordinary people admit the difference between the phenomenology of leeway freedom and the phenomenology of epistemic uncertainty. If they judge that their leeway phenomenology is understood as epistemic uncertainty, compatible with determinism, then we get evidence for the compatibilist view. In both condition 1 and condition 2, participants were told that they had a chance to get five cents if they pressed the correct button and considered what it was like to decide which option to choose. Two buttons, H and V, were shown at the bottom of the screen, and participants were not told which was the bonus button until after they had answered the compatibility question. In condition 1, like study 2, participants were asked to demonstrate their level of agreement with the statement that their phenomenology of deliberation includes leeway freedom on a 7-point scale. On the other hand, in condition 2, participants were asked to demonstrate their level of agreement with the following statement about epistemic uncertainty on a 7-point scale.

When wondering which option I'll choose, it feels like I don't know for sure before I select a button which button is the bonus button.

Only those participants who agreed with the above statements moved on to the comprehension test section, and only those who passed this test moved on to the compatibility question step. In condition 1, similar compatibility questions as in study 2 were shown.^21^ On the other hand, in condition 2, participants were asked whether to agree with the following statement on a 7-point scale.

If causal completeness is true, then I knew for sure before I selected a button which button was the bonus button.

The result of this experiment was that the mean value of the answers to the compatibility question in condition 1 was 5.34, *t*_(40)_ = 4.54, and *p* < 0.001. On the other hand, the mean of the answers to the compatibility questions in condition 2 was 2.66, *t*_(37)_ = –5.23, and *p* < 0.001.^22^ This result suggests that participants were incompatibilists about leeway freedom in the phenomenology of deliberation while they were compatibilists about epistemic uncertainty in the phenomenology of deliberation.^23^ Thus, it can be argued that participants do not judge leeway freedom in the phenomenology of deliberation as a kind of epistemic or doxastic possibility, which is compatible with determinism.

### 3.2 Three limitations

Study 2 by Deery et al. supports that, in contrast with the experiment by Nahmias et al., the non-philosophers' experience of leeway freedom in deliberation is incompatible with determinism not only in the case of imaginary deliberation but also in the case of actual deliberation. Moreover, the result of Deery et al. study 3 suggests that leeway freedom in the phenomenology of deliberation is considered not a kind of epistemic or doxastic possibility, and thus not in a compatibilist way.

Should we, then, conclude, following Deery et al. rather than Nahmias et al., that folk experiences of deliberation are incompatibilist? It would be fair to say at least the experiments conducted by Deery et al. included the procedures to help participants understand determinism, which was missing in the experiment conducted by Nahmias et al. The experiments by Deery et al., however, have at least three limitations. First, in their experiments, participants were asked to recall their experiences of deliberation after an introduction to the definition of determinism. It might therefore be the case that they cannot recall their experiences of deliberation without being influenced by the salience of determinism (cf. Bayne, 2017, pp. 636–637). For example, when participants were asked whether or not leeway freedom they experienced was compatible with determinism, they might be too uneasy about determinism to give a compatibilist answer because the concept of determinism might be salient in their minds in virtue of the act of asking, and thus the threshold for having free will became higher.^24^ If this is the case, we do not get data about the original experience of deliberation. Instead, we only get data about the experience of deliberation overwhelmingly instigated by the idea of determinism, which might be different from the ordinary phenomenology of deliberation.

Second, since participants were asked to answer the determinism test with true or false, they might have been able to pass the test even if they did not have sufficient comprehension of determinism. For example, there may be a case where someone who does not understand determinism provides an arbitrary answer but nonetheless passes the test. Thomas Nadelhoffer et al. suggest that ordinary people have difficulty in understanding determinism, particularly regarding three factors related to misunderstanding: a belief in epiphenomenal bypassing, the conflation of determinism with fatalism, and the intrusion of indeterministic assumptions (Nadelhoffer et al., 2023, pp. 2518–2519).^25^ Epiphenomenal bypassing is the thesis that belief, desire, and other mental activities cannot be causally potent in a deterministic world. Since determinism is compatible with the fact that our mental activities can exert causal influence, determinism does not imply epiphenomenal bypassing. According to fatalism, it is logically or conceptually true that there are no alternative possibilities no matter what happens (cf. Van Inwagen, 1983; Rice, 2023). Determinism does not entail fatalism because alternative possibilities exist within a deterministic world if the state of the world or the laws of nature are different from the actual reality. Intrusion is defined as a mental process by which non-deterministic assumptions are falsely introduced into a deterministic scenario or situation. Since intrusion evokes indeterministic ideas, which are incompatible with determinism, it impedes a correct understanding of determinism. If someone has trouble in these three matters, it is likely that they misunderstand determinism, and thus their judgment about compatibilism may be flawed.

(Nadelhoffer et al. 2023, pp. 2520–2525) examine whether non-expert people are immune to the influences of these three factors and possess an accurate understanding of determinism. In one experiment, they presented participants with a deterministic scenario and a surface comprehension test.^26^ Subsequently, participants who had passed the test were divided into three distinct groups and asked to indicate their level of agreement with four statements about epiphenomenal bypassing, fatalism, and intrusion in a 7-scale, respectively. The result was that in both the epiphenomenal bypassing and fatalism conditions, about 95% of the participants positively responded to at least one statement, and in the intrusion condition, about two-thirds of participants exhibited a similar response.^27^ This result indicates a real threat that non-expert ordinary people do not understand determinism sufficiently. Probably, in order to investigate the folk phenomenology of deliberation, we require multiple comprehension questions designed to check participants' understanding of determinism, such as questions regarding epiphenomenal bypassing, fatalism, and intrusion.

Third, study 3, conducted by Deery et al., which investigates whether folk phenomenology of deliberation is epistemological or doxastic, is inconclusive. In study 3, participants were asked whether it felt like they did not know for sure before selecting a button which button was the bonus button. In this question, the focus is what characteristics of the option are, i.e., whether or not the button is a bonus. Those who espouse epistemic or doxastic compatibilist interpretation, however, do not claim that deliberators are uncertain about the characteristics of the option they choose. Rather, they claim that we are uncertain which option we choose in deliberation. According to them, for example, when we deliberate whether to buy a new book on sale or not, we are uncertain not about the characteristics of the book, e.g., whether it is interesting for us or how many pages it has, but rather about which option we choose. Therefore, in order to examine folk phenomenology of deliberation, we must focus not on which button is the bonus but on which button is chosen. Since study 3 does not investigate epistemic or doxastic compatibilist view sufficiently, it is not enough to conclude that folk phenomenology of deliberation is not thought of as epistemological or doxastic and thus compatible with determinism.

Although these experiments have at least three limitations, they nonetheless reveal interesting characteristics of the folk phenomenology of leeway freedom in deliberation. Current studies on folk intuitions about whether one can act of one's own free will under determinism yield mixed results. While some studies, such as (Rose and Nichols 2013) and (Nadelhoffer et al. 2020), support the view that most people have incompatibilist intuitions, other studies, such as (Murray and Nahmias 2014) and (Inarimori et al. 2024a), suggest that people tend to have compatibilist intuitions. The studies by Deery et al. appear to provide indirect support for those who argue that we have incompatibilist intuitions and that the folk concept of free will is not compatible with determinism, such as (Rose and Nichols 2013) and (Nadelhoffer et al. 2020). Although the fact that people's experience of deliberation is incompatibilist does not entail that the folk concept of free will is also incompatibilist, compatibilists still need to explain why phenomenological experience should be set aside or discounted. Moreover, these experiments about the phenomenology of deliberation examine only leeway freedom, leaving open the question of how ordinary people experience source freedom in deliberation. Hence, studies focusing on source freedom in phenomenology of deliberation are also expected to contribute to the broader debate concerning the folk concept of free will and its compatibility with determinism.

## 4 Discussion: general methodological problems

In previous sections, we have seen philosophical discussions where the central issue is the dispute between the compatibilist and incompatibilist interpretations of leeway and source freedom in the experience of deliberation. Furthermore, we reviewed empirical studies of the folk phenomenology of deliberation and discussed their three limitations with regard to particular experiment settings. In this section, from a more general perspective, we will examine four methodological problems for the project of studying the phenomenology of deliberation and draw some philosophical implications concerning the nature of free will.

### 4.1 Problem of heterogeneity

As we have seen above, philosophers' claims about the experience of deliberation have been heterogeneous. Some philosophers claim that leeway freedom experienced in deliberation is incompatibilist, while other philosophers claim that it is compatibilist in the classical or epistemological(doxastic) sense. There is also a conflict as to whether (not only leeway freedom but also) source freedom is experienced in deliberation and, if so, whether it is compatibilist or incompatibilist. Also, there is some heterogeneity in the folk reports of the experience of leeway freedom. For example, in (Deery et al. 2013, p. 135, pp. 138–139, p. 142) experiments, while most participants reported experiencing incompatibilist leeway freedom in deliberation, a minority of participants reported not experiencing any leeway freedom. In addition, the preceding experiments have only investigated leeway freedom, and thus source freedom was not examined. Therefore, it is an open question whether or not we experience source freedom during deliberation. Because there is a possibility that deliberators experience compatibilist or incompatibilist leeway (source) freedom, the phenomenology of deliberation might vary across them (cf. Bayne, 2017, p. 637).

All of this suggests that there is no such thing as *the phenomenology* of deliberation; rather, there are *phenomenologies* of deliberation that are diverse and heterogeneous. Perhaps different people have different phenomenologies; e.g., some have a compatibilist phenomenology while others have an incompatibilist phenomenology; some experience leeway freedom, others experience source freedom; some experience both, and others experience neither. Furthermore, the experience of deliberation might not always be uniform even for the same person. For example, someone may experience leeway freedom in some cases of deliberation, and source freedom in other cases. Moreover, there may be a case that she experiences both types of freedom or neither.

The problem of heterogeneity can be especially troublesome when we try to justify a philosophical view of free will, such as (in)compatibilism or the leeway (source) view, by appealing to the phenomenology of deliberation. Suppose, for the sake of argument, that there has always been incompatibilist leeway freedom in the experience of deliberation and that this leeway freedom must be incompatibilist. Then, we would have a reason to support the view that leeway freedom in our experience of deliberation is incompatibilist. For there are no experiences that count as evidence to support the contrary views, such as the view that we all always experience compatibilist leeway freedom in deliberation. However, if the experiences of our deliberations are as diverse as suggested, we no longer have a reason to support a theory that requires a certain universality or necessity for the phenomenology of deliberation, such as that we *all must* experience (in)compatibilist leeway(source) freedom. In general, the greater the diversity of experience is, the more unspecific a true statement about the properties of that experience can only be (cf. Kriegel, 2015, p. 27). This implies that, given the heterogeneity of the phenomenology of deliberation, we can only make some unspecific claims about it, such as the claim that *some* leeway or source freedom is *more likely to* be experienced in deliberation, which is not very informative when it comes to justifying a more particular philosophical view of free will, such as (in)compatibilism or the leeway(source) view.

If our phenomenology of deliberation is diverse, we cannot use our experiences to defend (in)compatibilism or the leeway (source) view unless we limit their universality or necessity. Hence, whether our experiences can be evidence for strong and informative theories of free will depends on whether the phenomenology of deliberation is heterogeneous.

### 4.2 Problem of cognitive penetration

Apparently, there is a stronger heterogeneity in the description of the experience of deliberation among philosophers than among laypeople. For instance, most participants in the experiment conducted by Deery et al. report incompatibilist leeway freedom in their experience of deliberation, while philosophers' descriptions of their experience of deliberation appear to be more diverse. A possible explanation of this is that philosophers' descriptions is influenced, through the process of cognitive penetration,^28^ by their philosophical beliefs about free will; a philosopher experiences (in)compatibilist phenomenology of deliberation because of her doxastic commitment to an (in)compatibilist theory of free will. The heterogeneity in the description of the experience of deliberation among philosophers is, then, explained by the fact that different philosophers have different doxastic commitments about free will, which cognitively penetrate their experience of deliberation (cf. Nichols, 2012, p. 293).^29^

A problem that is caused by cognitive penetration is that it contributes to the problem of heterogeneity, which we discussed above. For instance, a philosopher's phenomenology of deliberation, influenced by her philosophical belief about free will, may be different from the phenomenology of another philosopher or a layperson who does not share her philosophical beliefs. That is, if cognitive penetration is a real phenomenon, the more variant philosophical views are, the more heterogeneous the phenomenology of deliberation will be.

Another problem is that if the experience of deliberation is cognitively penetrated by philosophical theories of free will, then it can be either viciously circular or methodologically redundant to justify a philosophical theory of free will by the experience of deliberation (cf. Nahmias et al., 2004, p. 165). Perhaps it is viciously circular to justify one's conception of free will (e.g., incompatibilist free will) by appealing to her (e.g., incompatibilist) phenomenology of deliberation, which is in turn cognitively penetrated by her (e.g., incompatibilist) doxastic commitment about free will. For, if experience is cognitively penetrated by philosophical theories, the epistemological status of these theories probably lies not in the phenomenology but in beliefs about theories since the content of the phenomenology depends on that of theories. Or, even if it is not viciously circular, it is methodologically redundant to appeal to one's phenomenology of deliberation in the context of justifying her theory of free will if it turns out that the phenomenology is the result of her belief about the theory. Suppose, for example, that someone proposes that free will can be understood as incompatibilist leeway freedom by appealing to her own experience of deliberation. Suppose also that her experience is cognitively penetrated by her belief that her theory is true. If this is the case, her phenomenology does not count as additional evidence for her theory. If we require evidence independent of philosophical theories, we should concentrate on the experiences of laypeople, which are less likely to be influenced by them.

In sum, cognitive penetration is problematic since it leads to the problem of heterogeneity. Moreover, cognitive penetration eliminates the need to appeal to the phenomenology of deliberation influenced by philosophical theories in investigating free will.

### 4.3 Problem of reliability

Philosophical and empirical studies of the phenomenology of deliberation rely on the assumption that we are able to describe our experience of deliberation reliably enough, at least in principle. However, this assumption of reliability can be challenged, especially when it comes to reporting the specific details of the experience of deliberation. For instance, when drinking a cup of coffee, it is not very difficult for us to report that we experience a coffee taste (rather than an apple juice taste). Unless we are professional tasters, however, it is difficult for us to report and describe the experiential details of the flavor of coffee. Analogously, it would not be very difficult for us to report that we experience a phenomenology of deliberation. Unless we are professionally trained introspectors, however, it would be difficult for us to report and describe the experiential details of the specific type of phenomenology of deliberation, including those details concerning whether the experience involves leeway freedom or source freedom as well as the ones concerning whether it involves compatibilist freedom or incompatibilist freedom. Perhaps we are not capable of reliably reporting and describing those details of our experience.

Moreover, there is a deeper problem about the specific details of the phenomenology of deliberation; i.e., it is not obvious that our phenomenology of deliberation really has those complex content concerning the leeway/source distinction, or the compatibilist/incompatibilist distinction in the first place (cf. Nichols, 2012, pp. 294–295; Bayne, 2017, pp. 634–635). It is a general philosophical issue whether one's experience can have highly sophisticated content in the first place. Sparse theorists about experiential content, who are skeptical about the very possibility of experience having highly sophisticated content, would argue that, for example, the alleged incompatibilist content of the phenomenology of deliberation, such as that we can do otherwise even if other conditions are exactly the same, or that we ourselves bring about action in a way that is not determined by other causes, cannot be experienced in principle. If experience cannot contain such complexity, then, all the reports of the experience of deliberation, by philosophers and by non-philosophers, are totally false and inaccurate; in short, they are complete confabulations.^30^

There are some other related reasons to be skeptical about the reliability of our report on the phenomenology of deliberation. For instance, the experience of deliberation can be confused with the belief about deliberation (note that the experience/belief confusion is not the same as cognitive penetration; e.g., it is possible that one confuses one's experience of deliberation with her belief or thought about deliberation, while her belief or thought has not causally influenced her experience of deliberation). The experience/belief confusion might explain why, for instance, philosophers talk about the specific details of their experience of deliberation while (assuming a sparse view about experiential content) their experience lacks those details as a matter of fact. A similar confusion can also occur among non-philosophers who do not have any theoretical beliefs about free will and deliberation. For instance, in Deery et al.s' experiment, participants were asked about what it was like to deliberate, and perhaps they were describing what they (non-experientially) thought during deliberation.

It is also possible that our report on the experience of deliberation is based on some problematic reasoning or interpretation. For example, Horgan states that there is a possibility of conflating (i) *not* experiencing their actions as determined by prior causes with (ii) experiencing their actions as *not* determined by prior causes (Horgan, 2015, p. 56).^31^ On the one hand, (i) is a statement that experience lacks a determinacy aspect. On the other hand, (ii) is a statement that experience does have an indeterminacy aspect. Also, Holton argues that the experience of our own choice not being determined by beliefs, desires, or intentions is misinterpreted as the experience of our own choice not being determined by anything (Holton, 2009, p. 417). Even if you experience that your beliefs, decisions, or intentions do not determine your choice, there is a possibility that other conditions, such as prior neurological states or environment, determine your choice. If these errors actually happen, it is difficult to accurately describe the experience of deliberation.

In addition to the problems above, two distinct compatibilist theories by Horgan and Deery raise some worries about the reliability of the report on the phenomenology of deliberation. Horgan concedes that, as we saw in Section 2.2, our free-agency phenomenology seems to include leeway and source freedom, but he denies the incompatibilist interpretation of the free-agency phenomenology, although it is seemingly self-evident. He thinks that the seeming incompatibilist report of the free-agency phenomenology is unreliable for several reasons. In addition to the occurrence of fallacious inference mentioned earlier, Horgan stresses that there is another misunderstanding factor: the hiddenness of the implicit contextual parameters. According to Horgan, the concept or judgment of the phenomenology of free-agency is implicitly governed by variable semantic context parameters, and the default setting on these parameters is compatibilist. The posing of the problem of free will and determinism, however, tends to be driven away from the default setting to a strong and incompatibilist setting (Horgan, 2015, pp. 56–58). This change in criteria is unnoticed because there are two kinds of content of experience—presentational content derived directly from phenomenology and judgmental content resulting from linguistic judgments about phenomenology— and the context parameters set by the judgmental content are not known by introspection (Horgan, 2015, pp. 56–58). Hence, when one engages in introspection in order to ascertain the question as to whether the content of the phenomenology of deliberation is compatibilist or not, they are implicitly inclined to judge that it is incompatibilist because the judgmental content is driven into a high-standard incompatibilist setting due to the very question they are seeking to answer. If Horgan's compatibilist strategy is indeed valid, we have difficulty in reliably describing the phenomenology of deliberation since the contextual parameter that determines its content is hidden from our introspection. This hiddenness might prevent us from describing it accurately.

Unlike Horgan, who assumes that the phenomenology of deliberation has only one genuine compatibilist truth condition, Deery counts on the idea that the phenomenology of deliberation has both libertarian and compatibilist contents (Deery, 2021, 2015a,b,c). If this is the case, this idea also complicates the interpretation of the introspective report on the phenomenology of deliberation.

In order to defend the dual-content view, Deery appeals to Chalmers' dual-content theory of (color) perception. This theory introduces two distinct forms of veridicality, and thus two different contents of perception of colors. On the one hand, a color experience associated with perfect veridicality is satisfied when the experienced object has perfect color as in Eden, the ideal world that exactly instantiates simple intrinsic qualities, which is supposed by color primitivism (Chalmers, 2006, p. 69). On the other hand, a color experience associated with imperfect or ordinary veridicality is satisfied when the experienced object has properties that match the perfect color, i.e., it normally causes a phenomenal color experience (Chalmers, 2006, pp. 69–70). Whether imperfect contents are satisfied depends on whether they play the role that perfect properties play in Eden properly. In this sense, perfect contents function as a regulative ideal to determine the standard for the veridicality of imperfect contents (Chalmers, 2006, p. 73).

Like Chalmers, Deery argues that the phenomenology of free agency has both perfect libertarian and imperfect compatibilist contents. The libertarian content is that (i) one is free to decide to do action A, (ii) the decision to do A is indeterministic^32^, and (iii) one is the source of A (Deery, 2021, p. 125). This content cannot be veridical when determinism is true: it can be true in agentive Eden where libertarianism, the view that we have free will which is incompatible with determinism, is true. In contrast, the compatibilist content is “[w]hatever feature (or set of features) ordinarily underpins free-agency phenomenology” (Deery, 2021, p. 127).^33^ According to Deery, the features relevant to the phenomenology of free agency are “the most sophisticated goal-directed behavior typically exhibited by human agents, often or even usually in the context of assigning moral responsibility” (Deery, 2021, p. 51). Since these behaviors can be performed in a deterministic world, the compatibilist content can be veridical even if determinism is correct. Of the two contents, the libertarian content is perfect, and the compatibilist content is imperfect. Hence, the libertarian content serves as the regulative ideal, which determines the appropriate matching relation for the compatibilist content. The compatibilist content, by contrast, is imperfect and gives the closest approximation to the ideal criterion from actions that are actually performed in the deterministic world. Therefore, even if determinism was true and thus the perfect libertarian content could not be satisfied, the imperfect compatibilist content could be veridical. If Deery's dual-content strategy is valid, it is true that we are justified to state the phenomenology of deliberation includes leeway freedom or source freedom. However, like Horgan's compatibilist strategy it introduces the complexity which inhibits us from capturing the phenomenology of deliberation adequately.

In sum, there are some theoretical issues about the reliability of reporting and describing the phenomenology of deliberation, including the problem of detailed content, the problem of experience/belief confusion, the problem of fallacious inference from experience, the problem of contextual variability, and the problem of double content. A further empirically elaborated approach is required to ascertain the reliability of self-reporting.

### 4.4 Problem of relevance

The phenomenology of deliberation has been considered to be closely related to free will because it includes the sense of leeway and source freedom. Indeed, deliberations themselves are also considered as paradigmatic instances of the exercise of free will. These statements are the basic suppositions behind the ongoing debate, as reviewed in this survey. Although these suppositions seem obvious, the significance of deliberation for the investigation of free will remains a matter of debate. For free will could be exercised without deliberating. Hence, whether deliberations are relevant for free will is an acute question.

In order to probe the relevance between free will and deliberation, we first look at the view that only actions resulting from serious deliberations involving multiple alternatives can be taken as exercises of free will. For instance, Campbell claims as follows:

Free will does not operate in these practical situations in which no conflict arises in the agent's mind between what he conceives to be his ‘duty' and what he feels to be his ‘strongest desire'. (Campbell, 1951, pp. 460–461)

According to Campbell, free will can be exercised only in situations where there is a need to deliberate whether to accomplish one's duty or follow their own desires. A similar idea is supported by van Inwagen.

...[T]here are at most two sorts of occasion on which the incompatibilist can admit that we exercise free will: cases of an actual struggle between perceived moral duty or long-term self-interest, on the one hand, and immediate desire, on the other; and cases of a conflict of incommensurable values (Van Inwagen, 1989[2017], p. 77).

These views that we cannot exercise free will unless we face serious deliberation were named “restrictivism” by (Fischer and Ravizza 1992).^34^

A consequence of restrictivism is that many ordinary actions have nothing to do with free will because acting out of a serious deliberation involving multiple choices is relatively rare in our everyday life.^35^ Van Inwagen (1989[2017]), p. 77) explicitly endorses this consequence.

Of course, there are objections to this view. (Van Inwagen 2017, p. 7) mentions an objection raised by Dennett that if restrictivism is a consequence of van Inwagen's argument, then this counts as a *reductio ad absurdum* for that argument. That is, since we exercise free will in everyday situations, if a particular view implies that we rarely exercise free will, then this view is hardly true. This point is also emphasized by Nahmias.^36^ (Nahmias 2006, p. 631) defines the situation required by restrictivism as a “close call” where “moments of (in)decision, which leave the agent, after deliberation, with nearly equally compelling alternatives for choice.” He then argues that free will can be exercised even when we do not face such a close call. The reason is that even if an agent does not face a close call and is thus confident about which options to choose, she can exercise free will because she can control her actions without being influenced by internal and external impediments (Nahmias, 2006, pp. 635–636, p. 659).

If Dennett and Nahmias are right and restrictivism is wrong, free will can be exercised not only in serious deliberations but also in other circumstances as well. This suggests that the phenomenology of deliberations about important matters is related to free will, but it is not all that matters. To focus only on the phenomenology of serious deliberation in the investigation of free will lead to miss the fact that we exercise free will in ordinary and easy situations.^37^

## 5 Conclusion

We have reviewed recent studies on the phenomenology of deliberation. We can draw the following lessons. First, a more precise approach should be taken to capture the phenomenology of deliberation. There are obstacles for people to describe and for experimenters to measure an experience, such as cognitive penetration, beliefs during deliberation, particular characteristics of the phenomenology of deliberation, and so on. Given these concerns, it is necessary to reconsider what is a clear and effective methods to capture the phenomenology of deliberation.

Second, since there is the problem of heterogeneity and the problem of relevance, the phenomenology of deliberation might not be a flawless remedy that would settle the problem of free will. Future research should aim to ascertain the extent to which our experience of deliberations is diverse. Moreover, if such diversity is confirmed, the factors influencing the heterogeneity of our experiences should be investigated. The phenomenology of deliberation is only one of the issues involved with free will. Nevertheless, investigating the phenomenology of deliberation provides clues to understand free will both philosophically and psychologically, and thus deserves more attention, as does moral responsibility.

Third, there is a need for an inquiry into the nature of the source freedom in the phenomenology of deliberation. Since it was incompatibilists who originally emphasized that importance of deliberation in free will and especially leeway freedom seems to be incompatible with determinism, they have been mainly focused on leeway freedom. For this reason, experiments on laypeople's phenomenology of deliberation have also focused on leeway freedom. However, source freedom is also an important element of the phenomenology of deliberation, so the exploration of this topic should contribute to understanding that phenomenology.

## Footnotes

1 There are at least two kinds of deliberation: practical deliberation and theoretical (or doxastic) deliberation (cf. Arpaly and Schroeder, 2014, p. 22). Although our explication of deliberation aims to capture a practical deliberation, if we modify it properly, we might get an explication of theoretical deliberation. That is, theoretical deliberation can be considered as the process of (i) excluding minor theoretical options in order to reduce unnecessary mental activity, (ii) trying to figure out the reasons for favoring or disfavoring theoretical options, and (iii) trying to decide the theoretical options to be believed or accepted.

2 The first element is derived from (Simon 1997, ch.5). The second and third elements are extracted from Coffman's (2017, pp. 590–591) survey paper on current works on deliberation.

3 We use the term “phenomenology” to mean the qualitative character of experience or consciousness, not the philosophical tradition or approach going back to Husserl.

4 Some current works deal with not the phenomenology of deliberation but with the phenomenology of agency (Bayne, 2008, 2017; Horgan, 2011; Deery et al., 2013; Pereboom, 2015; Nida-Rümelin, 2018; Clarke, 2019), the phenomenology of freedom (Kriegel, 2015), the phenomenology of free will (Nahmias et al., 2004; Holton, 2009; Horgan and Timmons, 2011), and the phenomenology of free agency (Horgan, 2011; Caruso, 2015; Deery, 2021; Strawson, 2020; Deery and Nahmias, 2023). Although these works seem to discuss related issues, we focus on the phenomenology of deliberation because the phenomenology discussed in these works can be mainly thought of as what is experienced in deliberation, and deliberation is a less theoretically-laden mental process, compared to agency, freedom, free will, and free agency. From this specific mental process of deliberation—especially its phenomenology, whose content might be that I have leeway or source freedom—we approach the more general concept of free will.

5 In this respect, there are problems called “etiological questions”: what is the generative and developmental process of the phenomenology of deliberation, and how does it influence our conception of free will (cf. Bayne, 2017, p. 633; Deery and Nahmias, 2023, p. 419)? We focus on and discuss the latter question, especially in Sections 4.2 and 4.4.

6 The leeway view and the source view are not mutually exclusive. Thus, one can state that both the leeway view and the source view are true without being contradictory.

7 In the contemporary philosophical discussion, this view can at least be traced back to Campbell.
Everyone must make the introspective experiment for himself: but I may perhaps venture to report, though at this late stage with extreme brevity, what I at least seem to find when I make the experiment myself. [...] I further find [...] that I cannot help believing that it lies with me here and now, quite absolutely, which of two genuinely open possibilities I adopt. (Campbell, 1951, p. 463).

8 Strictly speaking, determinism can be defined not only by states at any past time but also by states at any present or future time. For, laws of nature, especially the laws of physics, are symmetrical. That is, the laws of physics can determine how things proceed before and after an arbitrary time t. For the definition of determinism, see (Hoefer 2023) and (Vihvelin 2022).

9 This is a concise version of the consequence argument, which aims to show that leeway freedom is incompatible with determinism (cf. Van Inwagen, 1983).

10 Horgan calls leeway freedom “core optionality” (Horgan, 2015, p. 36).

11 Horgan denies this seeming verdict of the phenomenology of deliberation and argues that our action is in fact state-caused (cf. Horgan, 2011, 2015; Horgan and Nida-Rümelin, 2020).

12 There is also an eliminativist position according to which we do not experience source freedom at all (Dennett, 1984[2015], ch.3). What Dennett emphasizes is that we do not have experience whose content is that we have source freedom in deliberation, even though we can control our actions.

13 This clock consisted of a cathode ray oscilloscope and its spot of light.

14 In this experiment, some participants had preplanned to act in the near-future range of the clock before the act (Libet et al., 1982). The averaged time of readiness potentials (dubbed type I RPs) occurred about about 800-1000 ms prior to the action (cf. Libet, 2004, pp. 130-131). Even in these cases, W times remained similar to those in type II RPs. Are the forms of awareness in preplanning participants identical to the phenomenology of deliberation? Perhaps not, for even such preplanning does not contain the process of trying to weigh reasons and to decide among options. Rather, this forms of awareness might be related to preplanned decisions.

15 In condition 3 of the original experiment, in order to ascertain the impact of moral salience on the phenomenology, Deery and colleagues also presented participants with an actual decision between two charities, one of which has a higher moral significance (Deery et al., 2013, p. 137).

16 (Deery et al. 2013, p. 133) appealed to the following definition: “According to causal completeness, everything that happens is fully caused by what happened before it. This is true from the very beginning of the universe, so what happened next, and so on right up until the present. Causal completeness holds that everything is fully caused in this way, including people's decisions.” They used the technical term “causal completeness” in place of “determinism” so as to avoid undesired association (Deery et al., 2013, p. 131).

17 An example of descriptions was as follows (Deery et al., 2013, p. 133): “According to causal completeness, if we could somehow replay the entire past right up until St Helens erupted on May 18, 1980, then St Helens would once again erupt at that time. Another way to put this is to say that all the events leading up to the eruption made it so that the eruption had to happen.”

18 The following two questions were provided (the answer is “false” and “true”) (Deery et al., 2013, p. 133):
According to causal completeness, St Helens would have erupted on May 18, 1980 even if there had been no earthquake. According to causal completeness, if a week from now Barack Obama decides to have soda with dinner, all events leading up to that decision will make it the case that has to decide to have a soda with dinner.

19 Data were collected from 134 participants. 50 participants were assigned to condition 1 and 42 completed it. Of those, 38 reported phenomenology of the ability to do otherwise, and 35 passed the test of determinism. 48 participants were assigned to condition 2 and 42 completed it. Of those, 39 reported phenomenology of the ability to do otherwise, and 37 passed the test of determinism. 57 participants were assigned to condition 3 and 50 completed it. Of those, 43 reported phenomenology of the ability to do otherwise, and 40 passed the test of determinism (Deery et al., 2013, pp. 137–139).

20 The mean of the answer in condition 3 was 5.85, *t*_(39)_ = 7.66, *p* < 0.001 (Deery et al., 2013, p. 139).

21 In these questions, the phrase “even though” was omitted in order to avoid biased responses (Deery et al., 2013, p. 140).

22 In this experiment, data were collected from 91 participants. 53 participants were assigned to condition 1, and 47 completed it. Of those, 44 reported the phenomenology of leeway freedom, and 41 passed the test of determinism. Condition 2, on the other hand, began with 53 participants and 44 completed it. Of those, 39 reported phenomenology of epistemic uncertainty, and 38 passed the test of determinism (Deery et al., 2013, pp. 140–142).

23 A comparison of Condition 1 and Condition 2 by the *t* test showed that these conditions were significantly different from the midpoint of the scale, *t*_(76)_ = 6.85, *p* < 0.001 (Deery et al., 2013, p. 142).

24 The idea behind this argument depends on Horgan's contextualist theory of the phenomenology of deliberation (Horgan, 2015; Horgan and Nida-Rümelin, 2020). We review his idea in Section 4.3.

25 For a systematic review of comprehension errors and their consequences, see (Inarimori et al. 2024b).

26 This experiment (Study 1) was carried out with 397 participants and completed by 292 participants (Nadelhoffer et al., 2023, p. 2520).

27 Cronbach's alpha was 0.61 (epiphenomenal bypassing), 0.60 (fatalism), and 0.94 (intrusion) (Nadelhoffer et al., 2023, p. 2524).

28 Cognitive penetration is mainly discussed in the field of perception. For surveys on this topic, see (Stokes 2013) and (Silins 2016).

29 (Nahmias et al. 2004, p. 165) raise another possibility that there is a great diversity among philosophers with respect to experience of deliberation, and therefore theories about what experience of deliberation is like are also diverse.

30 If experience has a representational limit according to which experience cannot contain the theoretically complex structure, then there is also a limit to cognitive penetration since it requires that beliefs about complex theories can influence experience.

31 Later, Horgan withdraws this statement and argues that we, in fact, experience our own actions as not state-caused by acquainted experience while we misinterpret this experience as not state-caused at all (Horgan and Nida-Rümelin, 2020, pp. 290–291).

32 Indeterminacy here means an unconditional openness—one is free to decide A or to refrain from A-ing in unconditional sense (Deery, 2021, pp. 125–126).

33 Strictly speaking, Deery takes compatibilist content as “that one possesses or exercises whatever agentive features or capacities one typically does possess or exercise when one experiences acting freely, and that these features or capacities be suitably related to one's having the experience” (Deery, 2021, p. 127).

34 Strictly speaking, “restrictivism” is a abbreviation for “restrictive incompatibilism,” according to which incompatibilists must accept the inference rule (β) of the consequence argument, and this acceptance implies that we do not have significant free will in most cases (Fischer and Ravizza, 1992, p. 423).

35 Fischer and Ravizza argue that serious deliberations occur often (Fischer and Ravizza, 1992, p. 446). Whether serious deliberations occur rarely depends on the reference set of time spans under consideration. If we take into account all time spans, serious deliberations occur sometimes. However, if we focus on a single day, for example, serious deliberations are rare. Identifying the proper time span that is significant for the seriousness of deliberations is a pivotal question in the investigation for free will.

36 Nida-Rümelin emphasizes the same point (Nida-Rümelin, 2018, pp. 75–85).

37 (Bayne 2017, p. 634) distinguishes between the background phenomenology of freedom and the focal phenomenology of freedom. The background phenomenology of freedom is the feeling of being capable of exercising control over the physical and mental environment, and this feeling is supposed to accompany our everyday experiences universally. The focal phenomenology of freedom is the feeling of being able to settle matters that are currently open in contexts of deliberation and decision-making. To restate the debate on restrictivism on the basis of this distinction, we can argue that restrictivism focuses only on the focal phenomenology of freedom, but we should also consider situations in which the background phenomenology of freedom is involved.

## References

[B1] Arpaly N. Schroeder T. (2014). In Praise of Desire. Oxford: Oxford University Press. doi: 10.1093/acprof:oso/9780199348169.001.0001

[B2] Ayer A. J. (1954). “Freedom and necessity,” in Philosophical Essays (London: Palgrave Macmillan), 271–284. doi: 10.1007/978-1-349-00132-3_12

[B3] Bayne T. (2008). The phenomenology of agency. Philos. Compass 3, 182–202. doi: 10.1111/j.1747-9991.2007.00122.x

[B4] Bayne T. (2011). “Libet and the case for free will skepticism,” in Free Will and Modern Sciences, ed. R. Swinburne (Oxford: Oxford University Press), 25–46. doi: 10.5871/bacad/9780197264898.003.0003

[B5] Bayne T. (2017). “Free will and the phenomenology of agency,” in The Routlege Companion to Free Will, eds. K. Timpe, M. Griffith, and N. Levy (London: Routledge), 633–644.

[B6] Björnsson G. (2022). “Experimental philosophy and moral responsibility,” in The Oxford Handbook of Moral Responsibility, eds. D. K. Nelkin, and D. Pereboom (Oxford: Oxford University Press), 494–515. doi: 10.1093/oxfordhb/9780190679309.013.30

[B7] Campbell C. A. (1951). Is ‘freewill' a pseudo problem? Mind 60, 441–465. doi: 10.1093/mind/LX.240.441

[B8] Caruso G. (2015). Free will eliminativism: reference, error, and phenomenology. Philos. Stud. 172, 2823–2833. doi: 10.1007/s11098-015-0453-x

[B9] Castañeda H. (1975). Thinking and Doing. Dordrecht: D. Reidel Publishing Company.

[B10] Chalmers D. (2006). “Perception and the fall from Eden,” in Perceptual Experiences, eds. T. S. Gendler, and J. Hawthorne (Oxford: Oxford University Press), 49–125. doi: 10.1093/acprof:oso/9780199289769.003.0003

[B11] Clarke R. (1992). Deliberation and beliefs about one's abilities. Pac. Philos. Q. 73, 101–113. doi: 10.1111/j.1468-0114.1992.tb00330.x

[B12] Clarke R. (2019). Agent causation and the phenomenology of agency. Pac. Philos. Q. 100, 747–764. doi: 10.1111/papq.12275

[B13] Coffman E. J. (2017). “Deliberation,” in The Routledge Companion to Free Wil, eds. K. Timpe, M. Griffith, N. and Levy (London: Routledge), 590–599.

[B14] Coffman E. J. Warfield T. (2005). Deliberation and metaphysical freedom. Midwest Stud. Philos. 29, 25–44. doi: 10.1111/j.1475-4975.2005.00104.x

[B15] Deery O. (2015a). The fall from Eden: why libertarianism isn't justified by experience. Australas. J. Philos. 93, 319–334. doi: 10.1080/00048402.2014.968596

[B16] Deery O. (2015b). Why people believe in indeterminist free will. Philos. Stud. 172, 2033–2054. doi: 10.1007/s11098-014-0396-7

[B17] Deery O. (2015c). Is agentive experience compatible with determinism? Philos. Explor. 18, 2–19. doi: 10.1080/13869795.2013.874495

[B18] Deery O. (2021). Naturally Free Action. Oxford: Oxford University Press. doi: 10.1093/oso/9780198789796.001.0001

[B19] Deery O. Bedke M. Nichols S. (2013). “Phenomenal abilities: incompatibilism and the experience of agency,” in Oxford Studies in Agency and Responsibility, Vol. I, ed. D. Shoemaker (Oxford: Oxford University Press), 126–150. doi: 10.1093/acprof:oso/9780199694853.003.0006

[B20] Deery O. Nahmias E. (2023). “The experience of free agency,” in A Companion to Free Will, eds. J. Campbell, K. M. Mickelson, and V. A. White (Hobokn, NJ: Wiley-Blackwell), 417–433. doi: 10.1002/9781119210177.ch26

[B21] Dennett D. C. (1984[2015]). Elbow Room. Cambridge, MA: MIT Press.

[B22] Fischer J. M. Ravizza M. (1992). When the will is free. Philos. Perspect. 6, 423–451. doi: 10.2307/2214255

[B23] Grünbaum A. (1952). Causality and the science of human behavior. Am. Sci. 40, 665–676.

[B24] Haggard P. Eimer M. (1999). On the relation between brain potentials and the awareness of voluntary movements. Exp. Brain Res. 126, 128–133. doi: 10.1007/s002210050722 10333013

[B25] Hoefer C. (2023). “Causal determinism,” in The Stanford Encyclopedia of Philosophy (Summer 2024 Edition), eds. E. N. Zalta, and U. Nodelman. Available online at: https://plato.stanford.edu/archives/sum2024/entries/determinism-causal/ (Accessed January 9, 2026).

[B26] Holton R. (2009). Determinism, self-efficacy and the phenomenology of free will. Inquiry 52, 412–428. doi: 10.1080/00201740903087383

[B27] Horgan T. (2007a). Agentive phenomenal intentionality and the limits of introspection. Psyche 13, 1–29.

[B28] Horgan T. (2007b). Mental causation and the agent-exclusion problem. Erkenntnis 67, 183–200. doi: 10.1007/s10670-007-9067-9

[B29] Horgan T. (2011). The phenomenology of agency and freedom: lessons from introspection and lessons from its limits. Hum. Mente 15, 77–97.

[B30] Horgan T. (2015). “Injecting the phenomenology of agency into the free will debate,” in Oxford Studies in Agency and Responsibility, Vol. III, ed. D. Shoemaker (Oxford: Oxford University Press), 34–61. doi: 10.1093/acprof:oso/9780198744832.003.0003

[B31] Horgan T. Nida-Rümelin M. (2020). “On the satisfaction conditions of agentive phenomenology: a dialogue,” in The Routledge Handbook of Phenomenology of Agency, eds. C. Erhard, and T. Keiling (London: Routledge), 264–299. doi: 10.4324/9781315104249-23

[B32] Horgan T. Timmons M. (2011). Introspection and the phenomenology of free will: problems and prospects. *J. Conscious*. Stud. 18, 180–205.

[B33] Inarimori K. Haruki Y. Miyazono K. (2024a). Folk intuitions about free will and moral responsibility: evaluating the combined effects of misunderstandings about determinism and motivated cognition. Cogn. Sci. 48:e70014. doi: 10.1111/cogs.70014 39509582

[B34] Inarimori K. Honma S. Miyazono K. (2024b). Do we have (in)compatibilist intuitions? Surveying experimental research. Front. Psychol. 15:1369399. doi: 10.3389/fpsyg.2024.1369399 38711751 PMC11070465

[B35] Kapitan T. (1986). Deliberation and the presumption of open alternatives. Philos. Q. 36, 230–251. doi: 10.2307/2219771

[B36] Keller I. Heckhausen H. (1990). Readiness potentials preceding spontaneous motor acts: voluntary vs. involuntary control. Electroencephalogr. Clin. Neurophysiol. 76, 351–361. doi: 10.1016/0013-4694(90)90036-J 1699728

[B37] Kriegel U. (2015). The Varieties of Consciousness. Oxford: Oxford University Press. doi: 10.1093/acprof:oso/9780199846122.001.0001

[B38] Lehrer K. (1960). Can we know that we have free will by introspection? J. Philos. 57, 145–157. doi: 10.2307/2022533

[B39] Libet B. (1985). Unconscious cerebral initiative and the role of conscious will in voluntary action. Behav. Brain Sci. 8, 529–566. doi: 10.1017/S0140525X00044903

[B40] Libet B. (2004). Mind Time. Cambridge, MA: Harvard University Press. doi: 10.4159/9780674040168

[B41] Libet B. Gleason C. A. Wright E. W. Pearl D. K. (1983). Time of conscious intention to act in relation to onset of cerebral activities (readiness-potentials): the unconscious initiation of a freely voluntary act. Brain 106, 623–642. doi: 10.1093/brain/106.3.623 6640273

[B42] Libet B. Wright E. W. Gleason C. A. (1982). “Readiness-potentials preceding unrestricted ‘spontaneous' vs. pre-planned voluntary acts. Electroencephalogr. Clin. Neurophysiol. 54, 322–335. doi: 10.1016/0013-4694(82)90181-X 6179759

[B43] Moore G. E. (1912[2005]). Ethics. Oxford: Oxford University Press.

[B44] Murray D. Nahmias E. (2014). Explaining away incompatibilist intuitions. Philos. Phenomenol. Res. 88, 434–467. doi: 10.1111/j.1933-1592.2012.00609.x

[B45] Nadelhoffer T. Murray S. Murry E. (2023). Intuitions about free will and the failure to comprehend determinism. Erkenntnis 88, 2515–2536. doi: 10.1007/s10670-021-00465-y

[B46] Nadelhoffer T. Rose D. Buckwalter W. Nichols S. (2020). Natural compatibilism, indeterminism, and intrusive metaphysics. Cogn. Sci. 44:e12873. doi: 10.1111/cogs.12873 33145820

[B47] Nahmias E. (2006). Close calls and the confident agent: free will, deliberation, and alternate possibilities. Philos. Stud. 131, 627–667. doi: 10.1007/s11098-005-4542-0

[B48] Nahmias E. Morris S. G. Nadelhoffer T. Turner J. (2004). The phenomenology of free will. J. Conscious. Stud. 11, 162–179.

[B49] Nahmias E. Morris S. G. Nadelhoffer T. Turner J. (2005). Surveying freedom: folk intuitions about free will and moral responsibility. Philos. Psychol. 18, 561–584. doi: 10.1080/09515080500264180

[B50] Nahmias E. Morris S. G. Nadelhoffer T. Turner J. (2006). Is incompatibilism intuitive?' *Philos. Phenomenol. Res*. 73, 28–53. doi: 10.1111/j.1933-1592.2006.tb00603.x

[B51] Nelkin D. K. (2011). Making Sense of Freedom and Responsibility. Oxford: Oxford University Press. doi: 10.1093/acprof:oso/9780199608560.001.0001

[B52] Nichols S. (2012). The indeterminist intuition: source and status. Monist 95, 290–307. doi: 10.5840/monist201295216

[B53] Nichols S. Knobe J. (2007). Moral responsibility and determinism: the cognitive science of folk intuitions. Noûs 41, 663–685. doi: 10.1111/j.1468-0068.2007.00666.x

[B54] Nida-Rümelin M. (2018). Freedom and the phenomenology of agency. Erkenntnis 83, 61–87. doi: 10.1007/s10670-016-9872-0

[B55] Nolan D. (2009). “Platitudes and metaphysics,” in Conceptual Analysis and Philosophical Naturalism, eds. D. Braddon-Mitchell, and R. Nola (Cambridge, MA: MIT Press), 267–300. doi: 10.7551/mitpress/7507.003.0015

[B56] Nowell-Smith P. H. (1954). Ethics. London: Penguin Books.

[B57] O'Connor T. (1995[2003]). “Agent causation,” in Agents, Causes, and Events, ed. T. O'Connor (Oxford: Oxford University Press), 173–200. Reprinted in G. Watson (ed.) *Free Will*, 2nd Edn. Oxford: Oxford University Press, 257–284.

[B58] Pereboom D. (2014). Free Will, Agency and Meaning in Life. Oxford: Oxford University Press. doi: 10.1093/acprof:oso/9780199685516.001.0001

[B59] Pereboom D. (2015). “The phenomenology of agency and deterministic agent causation,” in Horizons of Authenticity in Phenomenology, Existentialism, and Moral Psychology: Essays in Honor of Charles Guignon, eds. H. Pederson, and M. Altman (Cham: Springer), 277–294. doi: 10.1007/978-94-017-9442-8_17

[B60] Rice H. (2023). “Fatalism,” in The Stanford Encyclopedia of Philosophy (Summer 2024 Edition), eds. E. N. Zalta, and U. Nodelman. Available online at: https://plato.stanford.edu/archives/sum2024/entries/fatalism/ (Accessed January 9, 2026).

[B61] Rose D. Nichols S. (2013). The lesson of bypassing. Rev. Philos. Psychol. 4, 599–619. doi: 10.1007/s13164-013-0154-3

[B62] Roskies A. L. (2011). “Why Libet studies don't pose a threat to free will,” in Conscious Will and Responsibility, eds. W. Sinnott-Armstrong, and L. Nadel (Oxford: Oxford University Press), 11–22. doi: 10.1093/acprof:oso/9780195381641.003.0003

[B63] Searle J. (1984). Minds, Brains and Science. Cambridge, MA: Harvard University Press.

[B64] Searle J. (2001). Rationality in Action. Cambridge, MA: MIT Press. doi: 10.7551/mitpress/5759.001.0001

[B65] Silins N. (2016). Cognitive penetration and the epistemology of perception. Philos. Compass 11, 24–42. doi: 10.1111/phc3.12292

[B66] Simon H. (1997). Administrative Behavior, 4th Edn. New York, NY: Free Press.

[B67] Soon C. S. Brass M. Heinze H.-J. Haynes J.-D. (2008). Unconscious determinants of free decisions in the human brain. Nat. Neurosci. 11, 543–545. doi: 10.1038/nn.2112 18408715

[B68] Soon C. S. He A. H. Bode S. Haynes J.-D. (2013). Predicting free choices for abstract intentions. Psychol. Cogn. Sci. 110, 6217–6222. doi: 10.1073/pnas.1212218110 23509300 PMC3625266

[B69] Stokes D. (2013). Cognitive penetrability of perception. Philos. Compass 8, 646–663. doi: 10.1111/phc3.12043

[B70] Strawson G. (1986[2010]). Freedom and Belief . Oxford: Oxford University Press.

[B71] Strawson G. (2020). “The phenomenology of free agency,” in The Routledge Handbook of Phenomenology of Agency, eds. C. Erhard, and T. Keiling (Londo: Routledge), 352–361. doi: 10.4324/9781315104249-28

[B72] Taylor R. (1964). Deliberation and Foreknowledge. Am. Philos. Q. 1, 73–80.

[B73] Trevana J. A. Miller J. (2002). Cortical movement preparation before and after a conscious decision to move. Conscious. Cogn. 11, 162–190. doi: 10.1006/ccog.2002.0548 12191935

[B74] Van Inwagen P. (1983). An Essay on Free Will. Oxford: Oxford University Press.

[B75] Van Inwagen P. (1989[2017]). “When is the will free?” *Philos. Perspect*. 3, 399–422. Reprinted in Van Inwagen P.Thinking about Free Will, Cambridge: Cambridge University Press, 60–80.

[B76] Van Inwagen P. (2017). Thinking about Free Will. Cambridge: Cambridge University Press. doi: 10.1017/9781316711101

[B77] Vihvelin K. (2022). “Arguments for Incompatibilism,” *The Stanford Encyclopedia of Philosophy (Fall 2022 Edition)*, eds. E. N. Zalta, andU. Nodelman. Available online at: https://plato.stanford.edu/archives/fall2022/entries/incompatibilism-arguments/

